# The Improvement of the Tribological Behaviour of Chemically Treated Abaca Fibre-Reinforced Polymer Composites

**DOI:** 10.3390/ma16247588

**Published:** 2023-12-10

**Authors:** Yucheng Liu, Yunhai Ma

**Affiliations:** 1 College of Agricultural Engineering, Jiangsu University, Zhenjiang 212013, China; 2 Key Laboratory of Bionic Engineering, Ministry of Education, Jilin University, Changchun 130022, China

**Keywords:** abaca fibre, chemical surface treatment, NaOH, silane coupling agent, polymer composite, tribological behaviour

## Abstract

Abaca fibres that have excellent mechanical properties are widely applied in the production and preparation of eco-friendly polymer composites as reinforcement materials. However, the weak interfacial bonding property of the abaca fibre and composite matrix limits the further extended application of abaca fibre-reinforced polymer composites. In this research, the findings demonstrate that, compared to raw abaca fibres, the interfacial shear strength (IFSS) value between the treated fibre and matrix is improved by 32% to 86%. Moreover, chemically treated abaca fibres could not only improve the wear resistance of the polymer composites, but also could promote the formation of primary and secondary plateaus. The best wear resistance behaviour was demonstrated by the sample with abaca fibres treated with 3% NaOH and 5% silane solutions, which had a maximum reduction in the sum wear rate of 28.44%. This research will provide detail on theoretical guidance and technical support for the development of eco-friendly natural fibre-reinforced polymer composites.

## 1. Introduction

Synthetic fibres, such as aramid, glass and carbon, are high-performance materials and have been widely applied in the production of automotive, civil engineering, naval, aerospace and wind power components for numerous decades [1,2,3,4]. However, they are less biodegradable, non-renewable and non-environmental resources and have caused problems ecological for the environment. Nowadays, the development of natural and recycled materials applied in preparation of fibre-reinforced polymer composites has attracted the attention of scientific researchers [5,6,7]. In fact, natural fibres have various specific characteristics and endow polymer composites with more excellent properties [8,9,10]. It is commonly recognized that the application of natural fibres to obtain polymer composites have significant economic and environmental benefits, and more new natural fibres provide a continuous supply of renewable raw materials for bio-polymer composite products. In general, natural fibres mainly include plant (cellulose), animal and mineral fibres [11,12].

Abaca fibre, as one of the important plant (cellulose) fibres, is derived from leaf sheathes of the abaca plant (*Musa textilis* Nee), and has excellent mechanical properties along with several other properties like rot and salt water resistances and high flexibility and durability [13,14]. It is considered that the mechanical strength of abaca fibre is the best among all natural fibres, and its tensile strength is even higher than that of its synthetic counterparts (like nylon) [15]. At present, abaca fibre-reinforced polymer composites have been applied in different automobile components.

However, abaca fibre, like other natural fibres, has some hydroxyl and other polar groups on its surface, which results in a decrease in the interfacial bonding property of natural fibre with the hydrophobic matrix [16,17]. In general, the surface treatment of abaca fibre should be performed before its use as a reinforcement material. Alkali treatment and silane treatment are commonly used surface treatment methods; they induce a physical or chemical change on the surface of abaca fibres and then improve their wettability and interfacial interaction, which could promote a relatively well interfacial bonding property [18]. During the recent two decades, many studies have prepared chemically modified natural fibre-reinforced composites and evaluated their mechanical and tribological properties. Shalwan et al. [19] analysed the influence of the treatments of different types of natural fibres on the mechanical and tribological properties of several thermoset and thermoplastic polymers. They found that the NaOH chemical treatment was the most useful treatment method for enhancing the interfacial adhesion of the natural fibres with the matrix, and then improved the mechanical and tribological properties of the polymers. Gang et al. [20] developed polyimide composites containing wood fibre treated with a coupling agent and suggested that the treatment with a coupling agent could enhance the interfacial adhesion between wood fibre and PI resin, and it played a very important role in improving the tensile and tribological properties of the carbon/PI composite. Siy et al. [21] reported a successful surface modification of the abaca fibres using two organotrialkoxysilane coupling agents. Moreover, they also concluded that hydrophobized abaca fibres could prevent high moisture absorption and improve the fibre dispersion and fibre/matrix bonding properties. In our previous study [22], abaca fibres were treated with 3 wt% NaOH solution, and then the tribology properties of the friction composites were studied systematically. NaOH-treated abaca fibres were beneficial to the improvement of the interfacial bond property of the fibre/matrix and the wear resistance property of the composites. As outlined in the section above, both the alkali treatment and silane treatment could enhance the interfacial bond property of the fibre and matrix and then improve the overall performances of the abaca fibre-reinforced composites. However, only a few studies investigated the influence of alkali-silane treatments on the tribological behaviour of eco-friendly abaca fibre-reinforced polymer composites.

In this work, the novelty of this research and its aim is to prepare chemically treated abaca fibres to satisfy the performance requirements of reinforcement materials and to develop eco-friendly natural fibre-reinforced polymer composites that are suitable for automotive friction parts. Abaca fibres were subjected to the NaOH treatment with various concentrations (1 wt%, 3 wt%, 5 wt% and 7 wt%), and were then treated with a 5 wt% silane coupling agent solution. The interfacial shear strength (IFSS) between the natural fibres and composite matrix was investigated. Moreover, raw and chemically treated abaca fibre-reinforced polymer composites were prepared with wet granulation technology, and then the influence of the different surface treatments on the tribological behaviour of the polymer composites was systematically evaluated. Finally, the wear mechanism of the abaca fibre-reinforced polymer composites was also discussed and analysed based on its worn surface morphology observations. We expected the newly developed chemically treated abaca fibres could exhibit better potential properties, and modified fibre-reinforced polymer composites could present excellent tribological behaviour and then could be a potential replacement for synthetic fibre-reinforced polymer composites.

## 2. Experimental Part

### 2.1. Materials

The abaca fibre-reinforced polymer composites mainly consist of the following components: *viz.* raw or modified abaca fibre, mineral composition fibre, phenolic resin, vermiculite powder, porous iron powder, precipitated barium sulphate, petroleum coke, flake graphite, alumina powder and frictional powder. The selection of raw materials for polymer composites was based on our previously published paper [23], and some of them were replaced. Phenolic resin, as a polymer, was purchased from Jinan, China. Abaca fibres were purchased from Guangzhou, China. Mineral composition fibre, porous iron powder and petroleum coke were purchased from Chaoyang, China. Vermiculite powder was purchased from Shijiazhuang, China. Precipitated barium sulphate was purchased from Hengshui, China. Flake graphite was purchased from Shanghai, China. Alumina powder was purchased from Zibo, China. Frictional powder was purchased from Jiaxing, China. Corn starches were obtained to prepare corn starch paste as liquid phase (liquid-containing binder) in wet granulation process. Silane coupling agent was obtained from Jinhui Chemicals, Jinan, China. Sodium hydroxide and absolute ethyl alcohol (analytically pure) were obtained from Beijing Chemicals, Beijing, China.

### 2.2. Surface Modification of Abaca Fibres

**Figure 1:**
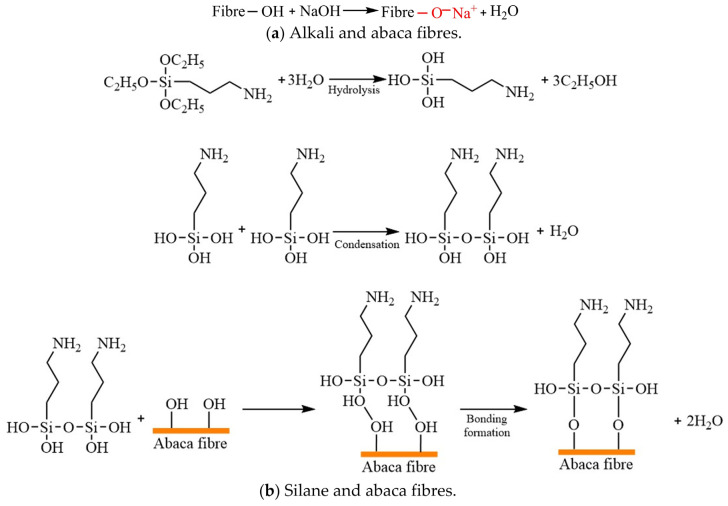
Interaction mechanism.

Abaca fibres were washed by using ultrasonic wave cleaning for 30 min, and then they were aired in a drying oven. The clean and dry abaca fibres were soaked in chemical solutions containing 1 wt%, 3 wt%, 5 wt% and 7 wt% of sodium hydroxide for 40 min at room temperature. Next, they were constantly flushed until pH 7 was attained and aired in a drying oven at 90 °C for 5 h. The pH of the solution required regular inspection using litmus paper. Alkaline-treated abaca fibres were impregnated directly with a solution of absolute ethyl alcohol/water (75/25 by volume) with a 5% silane coupling agent for 1.5 h under continuous stirring and room temperature, and then they were cleaned repeatedly with distilled water. The chemically treated abaca fibres were aired at 90 °C for 5 h and stored in drying apparatus prior to the preparation of polymer composite samples. The surface treatment processes of the abaca fibres refer to our published paper [6]. The interaction mechanisms between chemical reagent and abaca fibre are shown in Figure 1 [6,17].

### 2.3. Fabrication of Polymer Composites

**Figure 2:**
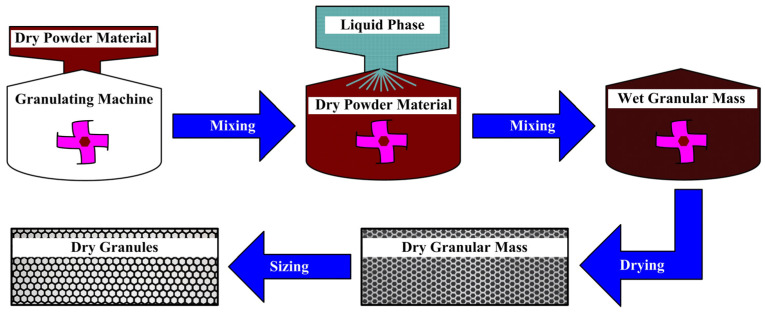
Schematic drawing of the wet granulation process.

**Figure 3:**
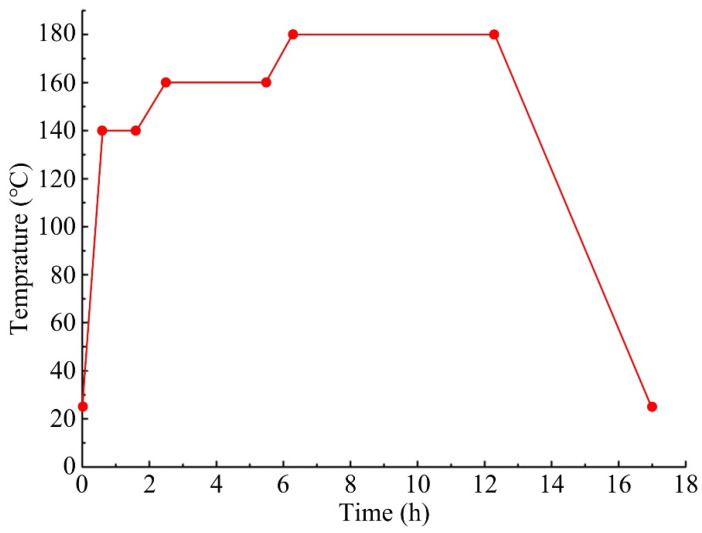
Thermal treatment process of the polymer composites.

All components of the polymer composites were thoroughly mixed using a blade-paddle mixer for 5 min at room temperature. After mixing all raw materials evenly, the mixture was placed into a granulating machine (JF805R, Jilin Wanda Mechanical Co., Ltd., Changchun, China) for wet granulation process. The schematic drawing of wet granulation process is shown in Figure 2 [23]. Wet granular masses were dried in an oven, and then dry granular masses (grain diameter: 1 mm–10 mm) were screened out. Next, they were compression moulded using a thermocompressor (JFY50, Jilin Wanda Mechanical Co., Ltd., Changchun, China) for 30 min, and then the samples were post-cured in a thermal treatment tank. Detailed thermal treatment process is illustrated in Figure 3. Finally, the polymer composite samples after heat treating process were machined into rectangular blocks with specified size of 25 × 25 × 6 mm^3^ for the following tribological performance tests. The preparation process of the polymer composites refers to our published paper [23].

The abaca fibre-reinforced polymer composite samples were labelled as PC1, PC3, PC5 and PC7. The letters “PC” refer to “Polymer Composite” and the number is the sodium hydroxide concentration. For comparison, the polymer composite with raw abaca fibres was prepared as the reference (Ref.).

### 2.4. Single Fibre Pull-Out Test

**Figure 4:**
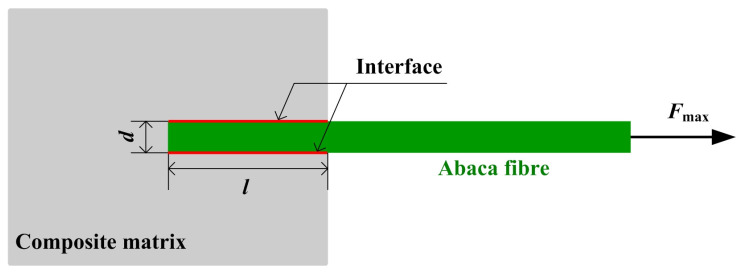
Schematic of single fibre pull-out test.

Interfacial shear strength (IFSS) between the natural fibre and composite matrix could usually be determined by a single fibre pull-out test [24]. In this work, the test was carried out on a microcomputer electronic universal tensile testing machine (DN-10 KN, Zhejiang Dana Automation Technology Co., Ltd., Huzhou, China) at a cross head speed of 1 mm/min. The detailed preparation of the test samples for this test was described in Ref. [25]. The schematic of a single fibre pull-out test is presented in Figure 4. Four tests were performed for each condition of chemical treatment. The IFSS value was calculated with the following equation:

(1) $$\tau_{\mathrm{IFSS}}=\frac{Failure\;\;\;\;\;\;load}{Interfacial\;\;\;area}=\frac{F_{\max}}{\pi \times d\times l},$$

where *F*_max_ is the maximum force recorded by the load cell, *d* is the diameter of the abaca fibre and *l* is the embedded abaca fibre length in the composite matrix.

### 2.5. Tribological Characterization

#### 2.5.1. Friction and Wear Properties

**Figure 5:**
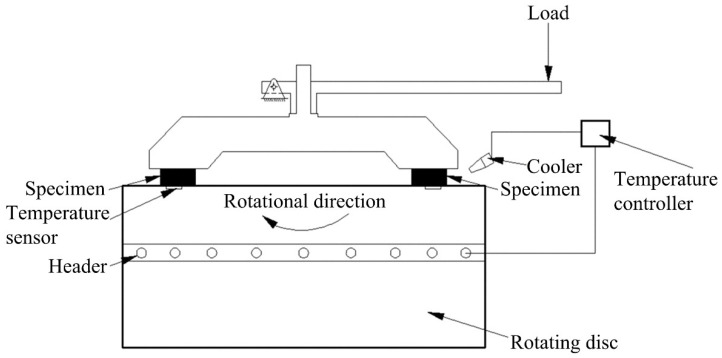
Equipment diagram of the friction-wear characteristic testing machine.

A friction-wear testing machine (JF150D-II, Jilin Wanda Mechanical Co., Ltd., Changchun, China) was applied to conduct friction-wear tests of samples Ref., PC1, PC3, PC5 and PC7 in accordance with the Chinese National Standard GB 5763-2018 [26], as shown in Figure 5. A cast iron (HT250) was chosen as materials of rotating disc, the rotating disc was used for counter pairs in the friction-wear testing machine, and it was operated with a constant speed of 480 rpm and heated with a regularly changing temperature. The working pressure on the polymer composites always fixed at 0.98 MPa. For all samples, the friction-wear test was repeated three times. Detailed experimental procedures are reported in our published papers [17,23].

During the fade test process, the prepared samples were evaluated at six consecutive rising temperatures of 100 °C, 150 °C, 200 °C, 250 °C, 300 °C and 350 °C. When each test ended at each test temperature, the weight of the testing samples was weighed by using a precision balance with accuracy of 0.001 g, and the thickness was measured by using a micrometre screw gauge with accuracy of 0.1 cm.

During the recovery test process, the prepared samples were evaluated at five successive decreasing temperatures of 300 °C, 250 °C, 200 °C, 150 °C and 100 °C. At each test temperature, the rotating disc was rotated for 1500 revolutions.

Friction coefficient (*μ*) and wear rate (*V*, cm^3^/N·m) of samples Ref., PC1, PC3, PC5 and PC7 were calculated with the following equations:

(2) $$\mu =\frac{f_{m}}{P},$$

(3) $$V=\frac{1}{2\pi r}\times \frac{A}{n}\times \frac{d_{1}-d_{2}}{f_{m}},$$

where *f_m_* is mean force of friction during the testing process (N); *P* is working pressure (N); *r* is centre distance of the rotating disc and sample (mm); *A* is total surface area of the contact surface with the rotating disc of the sample (mm^2^); *n* is total revolutions of the rotating disc (*n* = 5000); *d*_1_ and *d*_2_ are mean thickness of the tested samples before and after the friction/wear tests (cm), respectively.

#### 2.5.2. Fade and Recovery Properties

Fade ratio (*R*_fade_) and recovery ratio (*R*_recovery_) were defined using the following two formulas [27], respectively.

(4) $$R_{\mathrm{fade}}=\frac{\mu_{f_{350℃}}}{\mu_{f_{\max}}}\times 100\%,$$

(5) $$R_{\mathrm{recovery}}=\frac{\mu_{r_{100℃}}}{\mu_{r_{\max}}}\times 100\%,$$

where $\mu_{f_{350℃}}$ is friction coefficient at 350 °C and $\mu_{f_{max}}$ is maximum friction coefficient of the testing samples at the fade process; $\mu_{r_{100℃}}$ is friction coefficient at 100 °C and $\mu_{r_{\max}}$ is maximum friction coefficient at the recovery process.

Fade fluctuation (*F*_fade_) and recovery fluctuation (*F*_recovery_) of the polymer composite samples were defined using the following equations:

(6) $$F_{\mathrm{fade}}=\sum_{i=1}^{6}\frac{{\left({\bar{\mu }}_{f}-\mu_{i}\right)}^{2}}{6},i=6,$$

(7) $$F_{\mathrm{recovery}}=\sum_{j=1}^{5}\frac{{\left({\bar{\mu }}_{r}-\mu_{j}\right)}^{2}}{5},j=5,$$

where ${\bar{\mu }}_{f}$ is average friction coefficient at the fade process; *μ_i_* is friction coefficient at each specific experimental temperature at the fade process; ${\bar{\mu }}_{r}$ is friction coefficient during the recovery test; *μ_j_* is friction coefficient at each specific experimental temperature at the recovery process.

### 2.6. Characterization of Worn Surfaces

Wear surface morphologies of samples Ref., PC1, PC3, PC5 and PC7 were observed with a scanning electron microscope (SU3500, Hitachi Ltd., Tokyo, Japan) with a voltage of 15-kV. A gold coating was sprayed onto the worn surface of the samples using a sputter coater (SBC-12, KYKY Technology Co., Ltd., Beijing, China) prior to the observation.

## 3. Results and Discussions

### 3.1. Interfacial Shear Strength Analysis

The interfacial properties of natural fibres and composite matrices are one of the critical factors affecting the tribological properties of polymer composites. In this work, the interfacial properties were determined by measuring the interfacial shear strength (IFSS) generated in the fibre–matrix bonding area in a commonly used single fibre pull-out test. IFSS in the abaca fibre–composite matrix bonding area used for each raw, and treated abaca fibres are shown in Table 1; the treated abaca fibres have a higher IFSS with the composite matrix in comparison to the untreated abaca fibre. The IFSS values follow the order: F-PC3 ˃ F-PC5 ˃ F-PC7 ˃ F-PC1 ˃ F-Ref. The IFSS of the abaca fibre treated with 3% NaOH and 5% silane solutions exhibited a better interfacial property among all samples, and it was considerably higher than that of the untreated abaca fibre. The IFSS value of 14.56 MPa of sample F-PC3 was 86% higher than that of sample F-Ref (7.82 MPa). It has also already been reported in Refs. [25,28,29,30] that the proposed pretreatment could improve both the interaction and adhesion of the natural fibres with the composite matrix and then increase the interfacial shear strength of the natural fibre and composite matrix. In conclusion, the IFSS between the treated abaca fibre and composite matrix was better, resulting in a stronger reinforcing effect in polymer composites.

### 3.2. Frictional Stability Analysis

**Figure 6:**
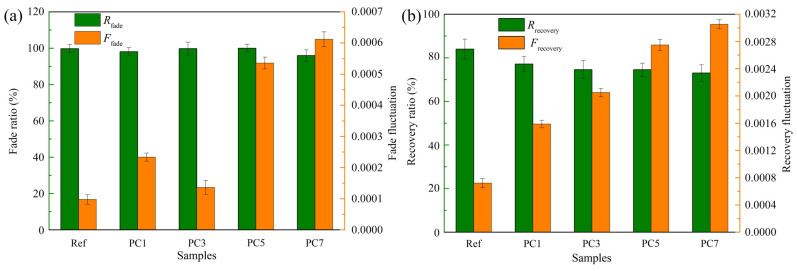
Effects of surface treatments on fade and recovery ratios; fade and recovery fluctuations of samples Ref., PC1, PC3, PC5 and PC7. (**a**) *R*_fade_ and *F*_fade_. (**b**) *R*_recovery_ and *F*_recovery_.

The friction test was conducted to evaluate the influence of different chemical treatments on the variation of the friction coefficient. In general, fade ratio, fade fluctuation, recovery ratio and recovery fluctuation are some of the parameters reflecting the frictional stability of polymer composites, and these test index results of samples Ref., PC1, PC3, PC5 and PC7 at the fade test process and recovery test process are shown in Figure 6.

Figure 6a presents the fade ratio (*R*_fade_) and fade fluctuation (*F*_fade_) of all tested samples during the fade test, calculated according to Equations (4) and (6). A value of 100% (*R*_fade_ value) means that the friction coefficient of the samples did not change with test temperature, which implies that the samples had an ideal fade resistance performance [31]. As shown in Figure 6a, the fade ratio of all tested samples was close to 100%, which indicates that they had excellent anti-recession performance during the fade process [32]. However, the fade fluctuation of all samples showed significant differences, their order was Ref. < PC3 < PC1 < PC5 < PC7. The *μ* variance was 9.6 × 10^−5^ for sample Ref., and its friction coefficient stability was improved more than those of the other four samples during the fade process, followed by sample PC3.

Figure 6b presents the recovery ratio (*R*_recovery_) and recovery fluctuation (*F*_recovery_) of all tested samples during the recovery test, calculated according to Equations (5) and (7). The *R*_recovery_ parameter is used to evaluate the composite’s capacity to gradually return to its nominal friction level after recovery test process. A value of 100% or higher indicates that the polymer composite has recovered or even exceeded its nominal friction level [31]. As presented in Figure 6b, sample Ref. demonstrated the best recovery performance and the least fluctuation among all samples, presenting a relatively good friction property at the recovery test process. Moreover, the values of *R*_recovery_ of the other four samples were above 70%, and this was an acceptable test result.

In general, sample Ref. had good fade resistance performance and recovery performance and low friction coefficient fluctuation, indicating that sample Ref. exhibited an excellent frictional stability during the fade test process and recovery test process. This may be because there is a contact plateau (Section 3.4) on the worn surface of the modified fibre-reinforced polymer composites during the friction process at an elevated temperature, resulting in a smaller friction coefficient, while the friction coefficients at low temperatures differ greatly. Then, the value is substituted into Equations (4)–(7), and the *R*_fade_ and *R*_recovery_ obtained are smaller, while the *F*_fade_ and *F*_recovery_ obtained are relatively larger. Although the chemical treatment of abaca fibres was not able to improve the friction stability of the prepared samples, the friction stability was in line with the requirements of GB 5763-2018. Compared to the other sample [27], all samples in this work also obtained better frictional stability.

### 3.3. Wear Performance Analysis

**Figure 7:**
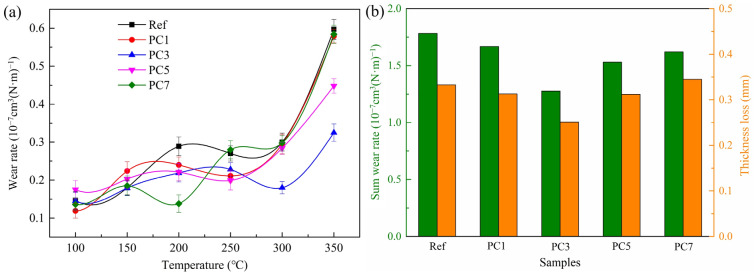
Wear resistance properties of samples Ref., PC1, PC3, PC5 and PC7: (**a**) wear rate; (**b**) sum wear rate.

In general, the wear resistance of polymer composites is mainly determined by the following parameters, e.g., test temperature, test load, component characteristic and transfer layer durability [32]. For polymer composites reinforcement materials and interfacial bonding property between the fibre and matrix play a decisive role in dominating wear performance [33]. The wear performance measurements were evaluated by means of wear testing at each test temperature, and the wear resistance property of the five abaca fibre-reinforced polymer composites was compared by evaluating their wear rate, sum wear rate and thickness loss, as shown in Figure 7.

Figure 7a clearly shows that the test temperature greatly affected the wear rate of the tested samples, and their wear rates were increased continuously along an increase in test temperature. This behaviour is in agreement with the previous studies [23,34]. At low temperature (100 °C–200 °C), the difference between the change of the wear rate of each sample was very small, and the fibre surface treatment did not greatly improve the wear resistance of the polymer composites. As the test temperature was raised, the trend of fluctuation phenomenon occurred at a set temperature of 250 °C and could be attributed to the decomposition of the phenolic resin and abaca fibre [35,36]. At elevated temperatures (300 °C–350 °C), the composite matrix accelerated decomposition and invalidation, weakened the shear strength of the samples and consequently caused the deterioration of the composites’ integrity and detachment of more matrix materials, which resulted in a rapid increase in wear rates. Moreover, it is worth noting that samples PC3 and PC5, with chemically treated fibres, present better wear resistance in comparison with sample Ref. which contained raw abaca fibres at elevated temperatures. Subsequently, the polymer composites were protected from wear [37]. The reason for better wear resistance is that a suitable surface treatment (appropriate chemical concentration) of abaca fibres effectively enhances the interfacial bonding property between the fibre and matrix (Table 1), and the treated fibres can effectively protect the integrity of the whole area of friction when high shear force acts on the friction surface of polymer composites during the test process, increasing the wear resistance of polymer composites. It has also already been reported in the published literature [38] that a good interfacial bonding property between the fibre and composite matrix leads to an improved wear resistance of the composites. In fact, excessively low and/or high sodium hydroxide concentrations are not conducive to strengthening the interfacial bonding property between the abaca fibre and matrix (Table 1), which ultimately leads to a relatively high wear rate of the tested samples.

Figure 7b presents that the sum wear rate and thickness loss of the polymer composites have the same variation trend. Samples with treated fibres provided a lower sum wear rate and thickness loss in comparison with sample Ref., and sample PC3 achieved the lowest sum wear rate and thickness loss among the treated fibre-reinforced composite samples; the sum wear rate was maximally lowered by 28.44% from 1.783 × 10^−7^ cm^3^/(N·m) to 1.276 × 10^−7^ cm^3^/(N·m). The possible reason for this is due to a good interfacial bonding property between the treated fibre and composite matrix in comparison with the raw fibre (Table 1), and a suitable concentration of the chemical solution for the abaca fibre surface treatment, absolutely reducing the separation and fracture of the treated abaca fibres during the friction process.

As a conclusion, the treated abaca fibres could enhance the wear resistance of the composite samples, particularly the abaca fibre treated with 3% NaOH and 5% silane solutions.

### 3.4. Friction and Wear Mechanism Analysis

**Figure 8:**
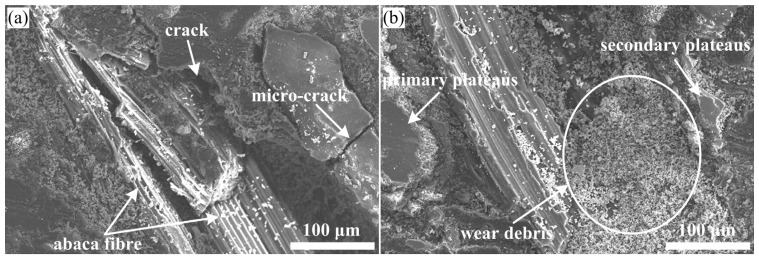
(**a**,**b**) Typical wear micrographs of Ref.

**Figure 9:**
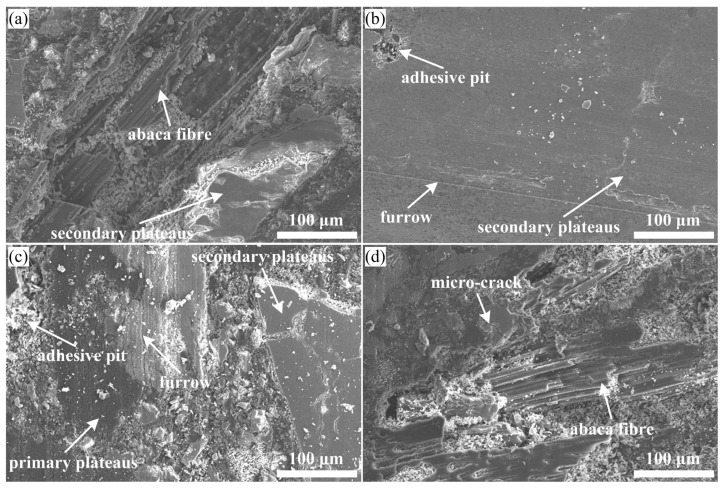
Typical wear micrographs of polymer composite worn surfaces. (**a**) PC1; (**b**) PC3; (**c**) PC5 and (**d**) PC7.

Worn surface morphologies (e.g., wear debris, crack, furrow, adhesive pit, primary and secondary plateaus, etc.) of the tested polymer composite samples could provide very critical indications on their friction and wear behaviours. To understand the effects of the chemical modification of the abaca fibre on the wear mechanism, worn surface morphologies were determined using SEM observation. The typical wear micrographs of the worn surface of samples Ref., PC1, PC3, PC5 and PC7 are displayed in Figure 8 and Figure 9.

**Figure 10:**
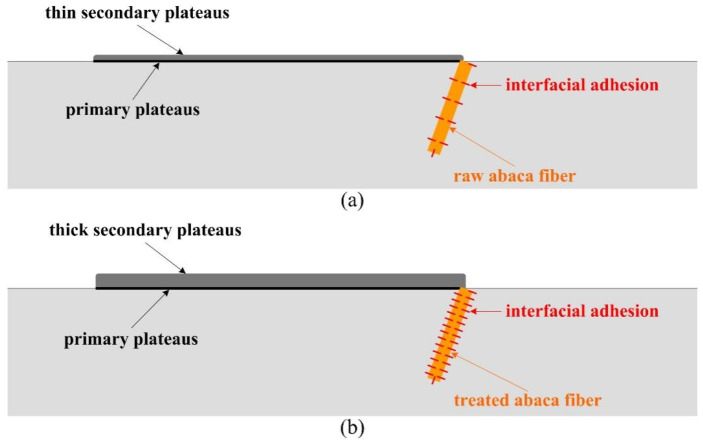
Schematic of the primary plateaus and secondary plateaus presented on the worn surface of the polymer composites. (**a**) Sample Ref. (**b**) Sample PC3.

Figure 8 displays the worn surface micrograph of sample Ref., which shows that the damage to the worn surface is serious with lots of wear debris, exposed fibre, broken fibre, split fibre, micro-cracks, adhesive pits, large areas of deep pits and a small amount of contact plateaus being present. Moreover, large cracks existed between the raw abaca fibre and matrix, indicating extremely weak interfacial adhesion, which easily causes the detachment of the fibre and matrix materials, and then leads to the appearance of pits on the worn surface of sample Ref. The above-observed results corresponded to the relatively high wear rate in Figure 7. In general terms, the impact of the interfacial bonding property on the tribological properties of the polymer composites is very significant, and thus a weak interfacial bonding property leads to poor tribological properties. Li et al. [39] also agreed that a weak interfacial bonding property between the fibre and polymer matrix was a challenge for large-scale applications of polymer composites in the field of friction materials. In this work, raw abaca fibres were covered by hydrophilic lignocellulosic molecules on their surface, and these molecules greatly affected the interlocking of the fibre and matrix, which eventually resulted in a loosened material matrix of sample Ref. during the friction process. Thus, a large number of base materials fell off under normal pressure and friction heat, and some hard asperities in the wear debris further damaged the composite matrix. Under this circumstance, stable primary plateaus were rarely present on the worn surface, which does not provide a nucleation point for the formation of secondary plateaus [17]. The thin secondary plateaus (Figure 10a) are not conducive to greatly enhance the wear resistance of sample Ref. All these morphological analyses are linked to the worst wear resistance or the highest wear rate (Figure 7). According to the above analysis, the main wear types of polymer composite samples with raw abaca fibres (Refs.) are adhesive wear and fatigue wear, as well as abrasive wear.

Figure 9a–d present the worn surface micrographs of the chemically treated abaca fibre-reinforced polymer composites, corresponding to samples PC1, PC3, PC5 and PC7, respectively. Compared to the worn surface of sample Ref. in Figure 8, the worn surfaces of the samples with treated abaca fibres were relatively smooth with different specifications of contact plateaus, especially samples PC3 and PC5. This is mainly attributable to the chemical surface modification of abaca fibres, which improves the interfacial shear strength of the natural fibre and composite matrix (Table 1). This is also consistent with the relatively low wear rate shown in Figure 7.

**Figure 11:**
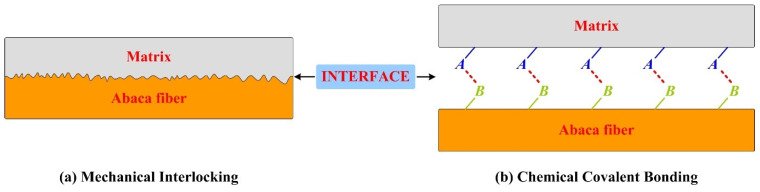
Interfacial bonding mechanisms of abaca fibre and polymer composite matrix.

Figure 9a shows that the worn surface of sample PC1 presents exposed fibre, fibre debonding, a fibre-shedding pit, wear debris and small areas of smooth surfaces. Moreover, some primary plateaus and secondary plateaus appeared on its worn surface. A possible reason for this is that the lower concentration of sodium hydroxide solution can only remove a small fraction of the non-cellulose components from the surface of the abaca fibre, which results in poor mechanical interlocking (Figure 11a), and the interfacial bonding property of the fibre and matrix is only improved by chemical covalent bonding (Figure 11b). Adhesive wear and abrasive wear are the main wear mechanisms of sample PC1.

Figure 9b,c show that the worn surfaces of samples PC3 and PC5 exhibit a slight furrow along the sliding direction and some adhesive pits. Particularly, it is clearly found that a dense structure and large area of contact plateaus exist on the worn surface, indicating a good interfacial bonding property of the abaca fibre and matrix. In general terms, the formation or deterioration of primary plateaus and secondary plateaus are mainly influenced by the reinforcing fibres in polymer materials [40]. Contact plateaus are usually divided into two types: primary plateaus and secondary plateaus. The abaca fibres treated with the proposed pretreatment that are bonded well to the composite matrix (Table 1), playing a crucial role in the formation of primary plateaus, which act as barriers to stop and gather wear particles, and then the primary plateaus serve as the nucleation points for the growth of secondary plateaus [23]. These accumulated wear particles were compacted on the surface of the primary plateaus, forming secondary plateaus [41]. Many relevant studies [42,43,44,45] showed that the presence of strong and compact secondary plateaus is very beneficial for the enhancement of the wear resistance of composites. In this work, relatively large secondary plateaus covered most of the area of the worn surfaces of samples PC3 and PC5, which is the primary reason for their lower wear rate and sum wear rate in Figure 7. The improvement of the wear-resisting property of samples PC3 and PC5 was associated with the fact that an appropriate concentration of sodium hydroxide solution and silane coupling agent solution could provide a better interfacial bonding property of the natural fibre and composite matrix, and then promote the formation of the thick secondary plateaus (Figure 10b). The formation of contact plateaus suggested that an adhesive wear mechanism acted on this polymer composite sample [46].

Figure 9d shows that the worn surface of sample PC7 presents exposed fibres, fibre-shedding pits, adhesive pits and micro-cracks, showing a poor interfacial bonding property of the fibre and matrix, which coincides with its friction and wear test results in Figure 7. A similar correlation between wear resistance and worn surfaces was also observed in the brake friction materials reinforced with calcium sulphate whiskers [47]. The abaca fibre in sample PC7 were treated with a higher chemical concentration; thus, chemical degradation and breakdown of the fibre did not provide enough mechanical strength, which ultimately resulted in a high wear rate for sample PC7.

In conclusion, the excellent properties of natural reinforcing fibres, as foundational materials, could withstand various forces (*viz*, shear force, normal force, impact force). In addition, they could then avoid fractures and excessive wear of the polymer composites during the friction process. Moreover, an appropriate chemical solvent and its concentration are critical for heightening the interfacial bonding property of the natural fibre and composite matrix, and thereby control the final tribological behaviour of the natural fibre-reinforced polymer composites.

## 4. Conclusions

The effects of the chemically treated abaca fibres on the tribological behaviour of the polymer composites was investigated and discussed in this work. It can be concluded from the findings that the eco-friendly natural fibre-reinforced polymer composites were successfully formulated from chemically modified abaca fibres, phenolic resins, property modifiers and space fillers. Compared to raw abaca fibres, the IFSS value between the treated fibre and matrix were improved by 32% to 86%. Moreover, the chemically treated fibres significantly improved the wear resistance of the polymer composites in comparison with that composite with raw abaca fibres. Sample PC3 achieved the lowest sum wear rate among the treated fibre-reinforced composite samples, and the sum wear rate was maximally lowered by 28.44%, from 1.783 × 10^−7^ cm^3^/(N·m) to 1.276 × 10^−7^ cm^3^/(N·m). This improved wear resistance response was mainly attributed to the better interfacial bonding property of the treated abaca fibre and matrix and large area contact plateaus. In conclusion, abaca fibre may be employed as a reinforcement material in eco-friendly polymer composites.

In future, physical and mechanical tests will be conducted to further evaluate the properties of polymer composites. Moreover, some advanced fibre treatment methods and composite preparation methods can be used to further improve the wear resistance of polymer composites.

## Figures and Tables

**Table 1:** IFSS between abaca fibre and composite matrix.

	F-Ref	F-PC1	F-PC3	F-PC5	F-PC7
IFSS	7.82 ± 1.21	10.36 ± 1.33	14.56 ± 1.75	12.95 ± 1.16	10.53 ± 1.58

## Data Availability

Data are contained within the article.
